# Research progress on the structure and function of endomucin

**DOI:** 10.1002/ame2.12142

**Published:** 2021-01-15

**Authors:** Guoxin Zhang, Xingjiu Yang, Ran Gao

**Affiliations:** ^1^ Key Laboratory of Human Disease Comparative Medicine (National Health and Family Planning Commission) Institute of Laboratory Animal Science Chinese Academy of Medical Sciences (CAMS) & Comparative Medicine Centre Peking Union Medical Collage (PUMC) Beijing PR China

**Keywords:** angiogenesis, endomucin, endothelial cells, related markers

## Abstract

Endomucin is a type I integral membrane glycoprotein, which is expressed in venous and capillary endothelial cells. It consists of 261 amino acids with an extracellular domain that is highly *O*‐glycosylated at serine and threonine residues and has several potential *N*‐glycosylation sites. Endomucin plays an important role in biological processes such as cell interaction, molecular cell signaling, angiogenesis and cell migration, and in recent years it has also been identified as an anti‐adhesion molecule and a marker of endothelial cells. While it has been shown to be involved in a number of physiological and pathological mechanisms, many of its functions remain unknown, and further study is needed. This article reviews research progress on the function of endomucin to date, in order to provide guidance for future studies.

## 1 INTRODUCTION

Endothelial cells rely on mechanical and humoral factors to maintain the endothelial barrier function and to avoid inappropriate loss of humor and solutes. Under physiological conditions, endothelial cells activate extracellular signals that lead to spatio‐temporal regulation of gene expression. Under pathological conditions such as inflammation, fluid permeability in the infected area is greatly increased, thus promoting host defense mechanisms.^1^ Therefore, studying the molecular mechanisms of endothelial cell‐specific regulation may reveal the cellular processes that lead to the occurrence and development of many diseases.

**FIGURE 1:**
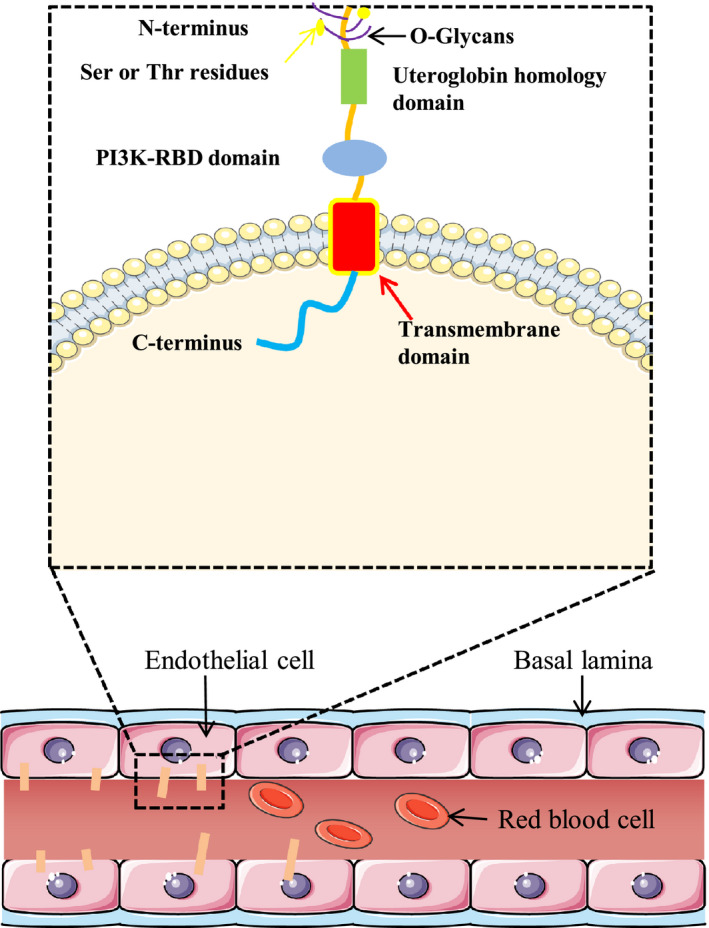
Schematic representation of transmembrane endomucin. This enlarged picture shows that the main structure of endomucin is divided into three parts, an extracellular domain (the N‐terminus with O‐glycans Ser or Thr residues and the PI3K‐RBD domain), an intracellular domain (the C‐terminus) and a transmembrane domain

**FIGURE 2:**
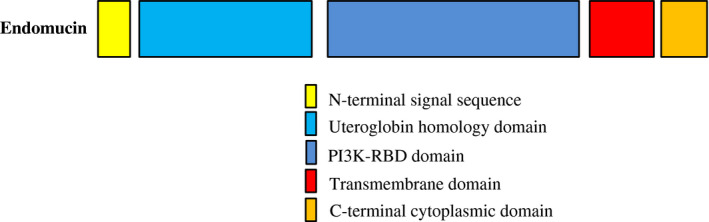
Structure of transmembrane endomucin. Endomucin is made of 261 amino acids with a molecular weight of 27.5 kDa and contains an N‐terminal signal sequence, a uteroglobin homology domain, a phosphatidylinositol 3‐kinase RAS‐binding domain (PI3K‐RBD), a transmembrane domain and C‐terminal cytoplasmic domain

Endomucin, a component of the glycocalyx, is a highly *O*‐glycosylated single transmembrane sialomucin expressed by endothelial cells, specifically by capillaries and venous endothelium, but not by most arterial endothelium.^2^, ^3^ Endomucin also exists in the lymphatic sinus endothelium, but not in the lymphatic endothelial cells of the subcapsular sinus.^4^ Mai Nguyen and others have established that the protein sequence of the long splicing variant of endomucin in humans and mice contains 261 amino acid residues and consists of a long extracellular part (aa 1‐C190), a transmembrane part (aa 191‐C214) and cytoplasmic sequence (aa 215‐C261) (Figures 1 and 2). In addition, 30% of its amino acids are serine or threonine residues, allowing it to covalently bind to *O*‐glycans through its extracellular domain.^5^, ^6^, ^7^

## 2 MAIN FUNCTION OF ENDOMUCIN

Endomucin is mainly expressed by endothelial cells around the postcapillary venules and a large number of highly vascularized tissues such as the heart, kidney and lung.^2^, ^5^, ^8^ Post‐capillary venules are the main site for leukocyte recruitment during internal perfusion. Endomucin inhibits leukocyte‐endothelial cell adhesion via lymphocyte function‐associated antigen 1 (LFA‐1)‐mediated binding to intercellular adhesion molecule 1 (ICAM‐1) in non‐inflamed tissues, and downregulation of endomucin by tumor necrosis factor (TNF)‐α critically promotes neutrophil infiltration into inflamed tissues.^9^ Treatment of endothelium with TNF‐α or the strong oxidant pervanadate leads to loss of cell‐surface endomucin and increases the levels of the C‐terminal fragment (EMCN‐CTF). Chemical inhibition of the cell surface protein ADAM10 alone or in combination with ADAM17 can block the release of EMCN‐CTF induced by TNF‐α. Thus endomucin represents a potential therapeutic target in the management of vascular inflammation.^10^

Endomucin is also expressed in retinal endothelial cells, and the expression level is reduced under high blood glucose in vitro and in vivo. Its overexpression can restore the glycocalyx of retinal endothelial cells induced by streptozotocin in diabetic rats.^11^ In addition to reducing the adhesion of leukocytes to endothelial cells, overexpression of endomucin can also reduce inflammation, stabilize the blood‐retinal barrier, and inhibit vascular leakage.^12^ Endomucin is the target of endothelial glycocalyx degradation; it protects diabetic patients from retinal vascular degeneration by restoring the glycocalyx of endothelial cells. Therefore, it represents a new treatment strategy for diabetic retinopathy.

Another mechanism by which endomucin enhances glycocalyx is through its interaction with galectin. Previous studies have found that *O*‐glycans on transmembrane mucins interact with galactose lectins to form multivalent protective crystal complexes, creating organized transmembrane mucins and aggregating on the glycocalyx on the cell surface.^13^, ^14^ Endomucin, also called endothelial sialomucin, interferes with the assembly of focal adhesion complexes and inhibits interaction between cells and the extracellular matrix, and maintains normal physiological functions.

### 2.1 The role of endomucin in angiogenesis

Studies on the expression of endomucin in the eye region have found that it inhibits retinal inflammation and promotes retinal microvascular angiogenesis during retinal development.^12^ Endomucin was recently identified as a novel vascular endothelial growth factor (VEGF)‐induced angiogenic regulator. Knockout of endomucin can reduce VEGF‐induced migration, proliferation and tube formation of human retinal microvascular endothelial cells in vitro, while its overexpression enhances these effects. Knockout of endomucin has also been shown to damage the vascularization of the developing mouse retina vascular in vivo.^12^

Recently, different sequences of the extracellular regions of endomucin have been studied in depth. The extracellular domain truncation mutants ∆21‐121 EMCN, reduced from ∆21‐161, is the minimal extracellular domain sufficient for VEGFR2‐mediated endothelial function. *N*‐Glycosylation of the EMCN extracellular domain is necessary for VEGFR2 function, but *O*‐glycosylation is not necessary for VEGFR2 internalization.^15^ D’Amore and others have also found that although VEGF stimulation promoted the endocytosis of VEGFR2 in endothelial cells, the knockout of endomucin prevented VEGFR2 endocytosis. Endomucin deletion led to increases in phosphorylation of VEGF after stimulation and total VEGFR2 protein. These results indicate that endomucin regulates the endocytosis and activity of VEGFR2 protein and is a potential clinical therapeutic target.^16^ Meanwhile, another study has reported a new role for endomucin as an effective angiogenesis regulator and points out its potential as a new therapeutic target for angiogenesis‐related diseases.^9^ In addition, endomucin indirectly regulates VEGFR2 angiogenesis through its extensive *O*‐glycolylation interactions with other carbohydrate‐binding proteins, such as different galactosin lectins. The key role of various galectins in angiogenesis has been demonstrated. For example, galectins‐1, ‐3, ‐8, and ‐9 have been shown to affect the angiogenesis process by binding to different endothelial cell surface receptors, activating different signaling pathways to regulate different events in the angiogenesis cascade.^17^ Many other findings suggest a role for endomucin in angiogenesis. The expression of endomucin is increased after endothelial cell proliferation or tumor conditioned media stimulation. GATA2 regulated endomucin gene expression is thought to be related to angiogenesis. Moreover, cystic embryoid bodies formed from VEGF‐deficient mouse embryonic stem cells contain endothelial cells that lack endomucin expression and cannot be organized into blood vessel‐like structures. The same study showed that endomucin is expressed by endothelial cells located downstream of VEGF.^18^ Future in‐depth studies of the relationship between endomucin and angiogenesis in tumor invasion and metastasis may suggest new targets for clinical anti‐angiogenesis therapy.

### 2.2 Effect of endomucin on biological functions of endothelial cells

Endothelial cell migration is the central link in the process of vascular morphogenesis.^19^ As is well known, the cytoskeleton is an important part of cell morphology and motor regulation. In endothelial cells with reduced endomucin expression, a decrease in F‐actin expression can be observed, which suggests that the presence of endomucin is a necessary condition for the formation of F‐actin in endothelial cells.^20^

Studies have shown that expression of endomucin is not affected by treatment with IL‐1 injection, but the level of fucosylation is significantly increased after IL‐1 treatment. Furthermore, endomucin glycosylation in endothelial cells is directly related to monocyte‐endothelial cell adhesion, which is achieved by blocking anti‐endomucin or sLex (sialyl‐Lewis X, sLex) antibodies in endothelial cells overexpressing FUT7 (α1,3‐karst‐based transfer Enzyme VII gene).^6^ In recent years, evidence has accumulated showing that endomucin is an anti‐adhesion molecule. Activation of factors stimulating endomucin can down‐regulate the expression of endomucin on the surface of endothelial cells and at the same time increase the adhesion between white blood cells and endothelial cells, while the absence of endomucin leads to an increase in the interaction between white blood cells and endothelial cells.^6^, ^9^

## 3 OTHER FUNCTIONS OF ENDOMUCIN

Endomucin was initially considered to be an endothelial cell‐specific protein, but it has also been shown to be involved in the development of embryonic stem cells.^21^ Endomucin has been shown to be a better marker for hematopoietic stem cells than CD34 protein. Human adult hematopoietic stem cells (HSCs) can be identified by hematopoietic progenitors associated with the expression of endomucin.^22^ Beside HSCs, endomucin can be also used as a marker in the study of skin endothelium.^3^

In terms of bone formation and development, the VEGF pathway controls the coupling of angiogenesis and osteogenesis in orthopedic implant‐bone integration by affecting the formation of CD31hi‐EMCNhi endothelial cells.^23^ Endomucin was found to be associated with rheumatoid arthritis in a Japanese population, and the endomucin allele associated with rheumatoid arthritis susceptibility may also be involved in the pathogenesis of rheumatoid arthritis.^24^ In terms of embryonic development, endomucin is expressed in the embryonic dorsal aorta and can inhibit cell adhesion. Immunohistochemistry revealed that endomucin was specifically expressed in dorsal aortic endothelial cells of E10.5 mouse embryos. The overexpression of endomucin strongly inhibited cell adhesion and aggregation, including in cultures of E10.5 dorsal aortic endothelial cells. These data suggest that endomucin plays a role in the separation of hematopoietic cells from endothelial cells in the early stage of hematopoiesis.^25^ The human endomucin molecule has no significant homology with any known glycoprotein. Based on sequence analysis, human endomucin may be involved in signal transduction. There are three protein kinase C phosphorylation sites and one casein kinase II phosphorylation site in the cytoplasmic tail, indicating that endomucin may act as a signaling molecule. In addition, there is some overlap in the RAS binding domain of the PI3K family.^26^ Human endomucin has also been shown to be consistent with certain motifs in the uteroglobin family. Uterine globin is a steroid‐induced multifunctional protein secreted by mucosal epithelial cells of the bronchus, uterus, and prostate. It is considered to be an effective anti‐inflammatory protein and may reverse malignant transformation.^27^, ^28^ When the differential genes in fast‐ and slow‐growing adenomas were confirmed by RNA sequencing analysis and qPCR, the results showed that the expression of endomucin was negatively correlated with tumor volume doubling time. Functionally, knocking down endomucin does not affect the migration of adenoma cells in vitro.^29^ Recently, Zhang et al showed that endothelial Notch signaling controls neutrophil trans‐migration via endomucin to modulate acute inflammation in hepatic ischemia reperfusion injury.^30^

## 4 OUTLOOK

Vascular endothelial cells have a very slow turnaround time, while tumor capillary endothelial cells undergo rapid proliferation and differentiation. Although there are many studies related to the role of endomucin in angiogenesis, it is unclear whether abnormal angiogenesis (such as angiogenesis in the growth of solid tumors) is related to the expression level of endomucin. Further studies confirming this role could provide a new molecular mechanism for abnormal angiogenesis and a new target for inhibiting the growth of abnormal blood vessels in clinical tumor therapy. Recently, a systematic analysis of the cancer genome database showed that combined detection of EMCN/MUC15 may be a potential prognostic marker for gastric cancer.^31^

At present, there are few studies on the adhesion or anti‐adhesion activity of endomucin. It has been reported that endomucin membrane protein is highly sensitive to L‐selectin oligosaccharides, and can be used as a ligand of L‐selectin to play a similar biological role.^32^ As a typical sialic acid mucin, endomucin is highly accessible on the cell surface, enabling some part of it to support or prevent cell adhesion. At the same time, endomucin is also a type of glycoprotein on the membrane. Its structural and expression level changes may affect the biological functions of cells such as invasion, metastasis, and adhesion. Whether endomucin affects the adhesion between tumor cells and endothelial cells has not been reported. The study of the adhesion between tumor cells and endothelial cells will provide a new theoretical basis for tumor metastasis.

## CONFLICT OF INTEREST

None.

## FUNDING INFORMATION

Chinese Academy of Medical Sciences Innovation Fund for Medical Sciences, Grant/Award Number: 2016‐I2M‐3‐019 and National Science and Technology Major Project, Grant/Award Number: 2017ZX10304402.

## AUTHOR CONTRIBUTIONS

Guoxin Zhang conceived and analyzed all the relevant data and wrote the manuscript. Xingjiu Yang helped during the literature research. Ran Gao conceived the review, corrected the manuscript several times and provided insights for the final version. All the authors read, contributed and agree with the content of the final version of the manuscript.
